# A systematic review of school-based weight-related interventions in the Gulf Cooperation Council countries

**DOI:** 10.1186/s13643-024-02475-7

**Published:** 2024-02-14

**Authors:** Mohammed Banany, Melissa Kang, Klaus Gebel, David Sibbritt

**Affiliations:** 1 School of Public Health, Faculty of Health, University of Technology Sydney, City Campus, PO Box 123, Broadway, NSW 2007 Australia; 2 Westmead Clinical School, Faculty of Medicine and Health, University of Sydney, Camperdown, NSW 2006 Australia; 3 School of Public Health, Faculty of Medicine and Health, University of Sydney, Camperdown, NSW 2006 Australia

**Keywords:** Weight-related interventions, Obesity, School-based interventions, Health promotion, Gulf Cooperation Council countries

## Abstract

**Background:**

The prevalence of childhood overweight and obesity has increased at alarming levels in the Gulf Cooperation Council (GCC) countries (Saudi Arabia, United Arab Emirates (UAE), Kuwait, Bahrain, Oman, and Qatar). Weight-related interventions are urgently required in these countries to tackle childhood overweight and obesity and their-related consequences. To date, no systematic review has synthesised school-based weight-related interventions in the six GCC countries. This study aims to systematically review school-based, weight-related interventions conducted in the GCC countries, investigating the intervention characteristics, components, and outcomes.

**Methods:**

Medline, Scopus, and ProQuest databases were searched for peer-reviewed literature published in English without date restriction and Google Scholar for grey literature using combined Medical Subject Heading (MeSH) terms and keywords under five relevant concepts including population, setting, interventions, outcomes, and geographical location. Following the Preferred Reporting Items for Systematic Reviews and Meta-Analyses (PRISMA), records were identified, screened for eligibility, and included in this review. Using the Effective Public Health Practice Project tool, the methodological quality of the included studies was assessed independently by two authors.

**Results:**

Out of 1303 initially identified records, eight peer-reviewed articles and three doctoral theses were included in this review. The age of the students in the included studies ranged between 5 to 19 years, and the sample sizes between 28 and 3,967 students. The studies included between one and thirty public and private schools. Of the included studies, six were randomised controlled trials, four pre-post studies and one used a post-study design. Only four of the eleven studies were theory based. The included studies reported various improvements in the students’ weight or weight-related lifestyle behaviours, such as healthier dietary choices, increased physical activity, and decreased sedentary behaviour.

**Conclusions:**

This review suggests the potential effectiveness of school-based interventions in the GCC countries. However, a thorough evaluation of these studies revealed significant methodological limitations that must be acknowledged in interpreting these results. Future studies in this field should be theory-based and use more rigorous evaluation methods.

**Systematic review registration:**

PROSPERO registration number: CRD42020156535.

## Background

The global prevalence of childhood obesity, defined here as affecting children and adolescents aged under 18 years [1], has substantially increased in recent decades. In 2016, over 340 million children and adolescents were considered either overweight or obese [2]. Childhood obesity is associated with obesity in adult life, which in turn adversely affects health [3–5]. It is also associated with multiple co-morbidities, including metabolic syndrome, type 2 diabetes, pulmonary, cardiovascular, and musculoskeletal complications [6–9]. Furthermore, social (e.g. discrimination) and mental (e.g. depression, low self-esteem, and negative body image) health issues are important consequences of obesity that predispose to poor quality of life [10–12].

In the past two decades the prevalence of childhood obesity has risen significantly among the Gulf Cooperation Council (GCC) countries (Saudi Arabia, the United Arab Emirates (UAE), Kuwait, Bahrain, Oman, and Qatar) and is considered among the highest in the world [13, 14]. In 2019, 18.4% and 12% of Saudi boys aged 6–16 years, were obese and overweight, respectively; compared to 18% and 14.2% of girls of the same age [15]. Al Yazeedi and Berry in 2019 [16], also reported that the average rate of combined overweight and obesity for boys aged 6–10 years in Saudi Arabia, Kuwait, and Emirates was 14.2% compared to 25% among girls.

Various strategies and interventions have been explored to address childhood overweight and obesity [17–19]. The implementation of multi-component school-based interventions, targeting diet, physical activity, and sedentary behaviour [20], is a common strategy used for addressing obesity among schoolchildren. Such strategies emphasise the integral role of schools as venues for health promotion and aligns with the broader literature, which consistently highlights schools as ideal settings for childhood obesity interventions [21–23]. In the context of obesity prevention interventions, “school stakeholders” refers to a diverse group, including school principals and teachers as well as students’ parents/caregivers, health professionals, government entities, and community organisations. These stakeholders collaborate to implement and support various aspects of obesity prevention initiatives, ranging from curriculum development to policy advocacy and program evaluation [24]. Moreover, science, physical education and senior management staff can facilitate discussions with students on health-related topics such as body image, nutrition, and weight control [25].

Multiple systematic reviews have examined school-based obesity interventions in other parts of world [21, 26–30], however, no such review has been conducted in the GCC countries. Two systematic reviews have investigated the prevalence of overweight and obesity in the Gulf countries [13, 31], one systematic review looked at interventions for obesity among adults [32], and another study reviewed physical activity interventions among people in the Arabic countries, where one third of the included studies targeted children and adolescents [33]. This systematic review adds value in the context of addressing childhood obesity, by specifically focusing on the GCC countries, where such reviews are lacking. The review’s findings can potentially inform and influence health promotion strategies and policies within the education systems of the GCC countries. In addition, these findings can guide policy decisions related to students’ dietary behaviours and practices, and physical activity types and duration in schools. Therefore, this systematic review aims to synthesise school-based weight-related interventions conducted in the GCC countries.

## Materials and methods

In 2020, the protocol for this review was registered with the International Prospective Register of Systematic Reviews (PROSPERO ID: CRD42020156535). Our reporting conforms to the Preferred Reporting Items for Systematic Reviews and Meta-Analyses (PRISMA) [34].

### Search strategy

A systematic search was conducted in November 2022 using the databases Medline, Scopus, and ProQuest, which were chosen due to their comprehensive coverage of medical and health sciences literature to retrieve all relevant peer-reviewed studies published in English. The search was conducted without date restrictions to capture the full extent of research conducted in this area, with the search being completed on 17 November 2022.

Combinations of Medical Subject Heading (MeSH) terms and keywords were used under five common concepts: (1) population [‘adolescence’ or ‘teen’ or ‘youth’ or ‘child’ or ‘student’], (2) setting [‘school’ or ‘school-based’], (3) interventions [‘intervention’ or ‘initiative’ or ‘program’ or ‘project’ or ‘physical’ or ‘exercise’ or ‘sedentary’ or ‘diet’ or ‘nutrition’ or ‘behaviour’], (4) outcomes [‘obesity’ or ‘weight’ or ‘body mass index’ or ‘BMI’], and (5) geographical location [‘Gulf’ or “Saudi’ or ‘Emirates’ or ‘Kuwait’ or ‘Bahrain’ or ‘Oman’ or ‘Qatar’ or ‘KSA’ or ‘UAE’ or ‘Arab’]. Google Scholar was searched for grey literature. All identified records were imported into EndNote version 9 (Clarivate Plc, Philadelphia, United States and London, United Kingdom).

In the initial screening phase, after the removal of duplicates, two authors independently reviewed the titles and abstracts of the remaining studies to exclude irrelevant records. To minimise potential bias and enhance the decision-making process, any discrepancies between the authors were resolved by a third author. Backward citation tracking was also used to identify any additional studies.

**Fig. 1:**
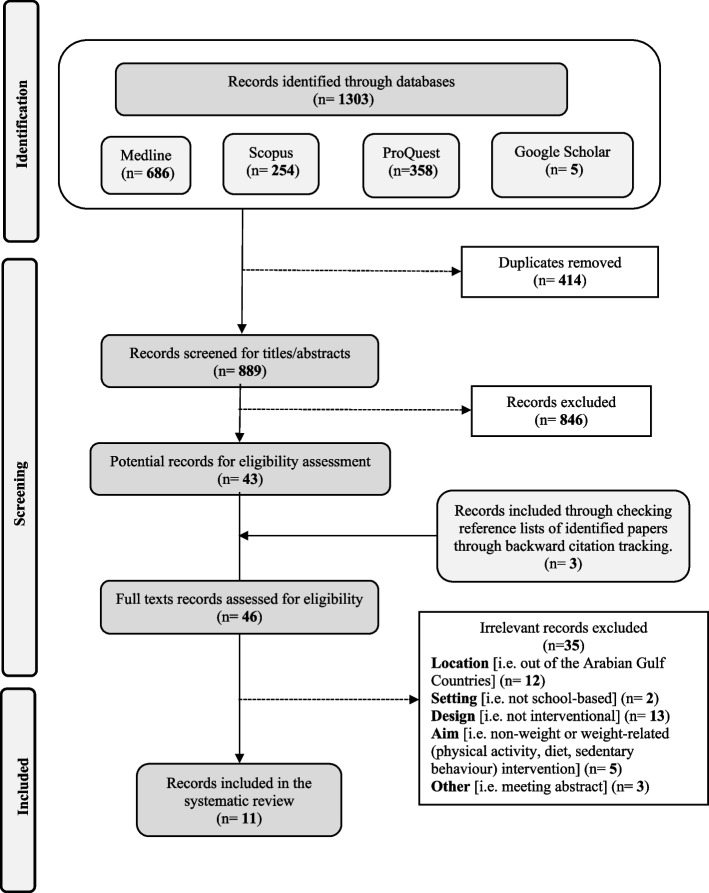
PRISMA flowchart for the included studies in this systematic review

As illustrated in Fig. 1, the initial search of the databases yielded 1303 records. After removing 414 duplicates, 889 records were screened based on their titles and abstracts, excluding 846 and leaving 43 potential records. These records were further screened based on their full-texts, and an additional three records were added through cross-referencing, yielding 46 studies. Based on the full text examination, 11 studies met the inclusion and exclusion criteria: 8 peer-reviewed studies [35–42] and three doctoral theses [43–45] were included in this systematic review.

### Inclusion and exclusion criteria

The full texts of all potential studies were assessed for their eligibility to be included in this review if they (1) were school-based interventions conducted in any of the GCC countries; (2) targeted school-aged students (5–18 years old) during school hours; (3) included at least one weight-related lifestyle intervention (physical activity, sedentary behaviour or diet); and (4) had at least one anthropometric measure of body weight or weight-linked lifestyle factor (physical activity, sedentary behaviour or diet) as a primary or secondary outcome of interest. However, a study was excluded if it was not conducted in any of the GCC countries, was not school-based, was not an intervention study, or if the outcome was not related to weight or weight-related lifestyle factors. A systematic review of correlates of, and interventions for weight and weight-related behaviours among adults in the Gulf countries [32] only identified seven interventions, four of these did not have control groups. Therefore, this review also included pre-post school-based intervention studies.

### Data extraction and synthesis

Data from each included study was extracted into a table that included study title, authors, publication year, country, study aim(s), design, participant characteristics, sample size, school characteristics, intervention components, duration, and outcomes. Due to the heterogeneity of the studies’ characteristics, intervention components and outcome measures, meta-analysis was not suitable and hence a narrative synthesis was conducted. The tabulated data were analysed to explore the study design, intervention components and outcomes of interest, and limitations of the studies. Driven by the “Behaviour Change Wheel” (BCW) [46], the intervention components included nine intervention functions, which are education; persuasion; incentivisation; coercion; training; enablement; modelling; environmental restructuring; and restrictions. Outcomes of interest were measures of students’ anthropometrics, diet, physical activity, and sedentary behaviour. For each particular outcome, the interventions were broadly categorised as effective, not effective, or mixed effects. An intervention was considered ‘effective’ if there was a statistically significant improvement concerning a particular outcome. An intervention was regarded as ‘not effective’ if the results showed a non-significant improvement or no change at all. If the results were mixed among a particular outcome (e.g. a significant reduction in the prevalence of obesity, but no improvement in the prevalence of overweight), the intervention was considered mixed in terms of the effectiveness.

### Quality assessment

Two authors independently assessed the quality of the included studies using the Effective Public Health Practice Project (EPHPP) quality assessment tool, which has six key quality components, including selection bias, study design, confounders, blinding, data collection methods, and withdrawals or dropouts [47, 48]. Each individual component was independently rated as strong, moderate, or weak. An overall quality assessment for each study was rated strong, moderate, or weak accordingly. The overall quality of each study was assigned ‘strong’ if at least 4 of 6 quality criteria were rated strong and no criterion was rated weak; assigned ‘moderate’ when only one quality criterion was rated weak; and assigned ‘weak’ if two or more quality criteria were rated weak (Table 3). To ensure consistency, the authors crosschecked the quality of the included studies. Discrepancies between the reviewers’ ratings was discussed until a consensus was achieved.

## Results

Characteristics of the studies, such as study design and duration, as well as the participants’ characteristics in terms of age, gender, sample size and school characteristics, are provided in Table 1.

**Table 1:** Characteristics of included school-based intervention studies (n = 11)

Author, year and country	Study characteristics	Study participants
Al-Failakawi (2017) [43] Kuwait	**Design:** RCT **Duration:** 3 months	**Age:** 14–18 years **Mean age ± SD:** NR **Sex:** Girls only **Sample size:** n = 128 **School/s:** 1 public secondary school
Al-Jaaly (2017) [35] Saudi Arabia	**Design:** Cluster RCT **Duration:** 1 month	**Age:** 13–15 years (82%) & 16 years (18%) **Mean age ± SD:** 14 years **Sex:** Girls only **Sample size:** n = 28 **School/s:** 2 (1 public & 1 private) intermediate schools
Allafi (2020) [36] Kuwait	**Design:** Pre-post study **Duration:** NR	**Age:** 9–11 years **Mean age ± SD:** NR **Sex:** Boys and girls **Sample size:** n = 225 (boys: n = 110 & girls: n = 115) **School/s:** 6 public primary schools
Al-Mughamisi (2021) Saudi Arabia [44]	**Design:** Pre-post study **Duration:** Participatory workshops (timeline not mentioned) followed by a 2-day pilot study	**Age:** 13–18 years **Mean age ± SD:** NR **Sex:** Girls only **Sample size:** n = 116 **School/s:** 2 public (1 intermediate & 1 secondary) schools
Bahathig & Abu Saad (2022) [37] Saudi Arabia	**Design:** Cluster RCT **Duration:** 2 months	**Age:** 13–14 years **Mean age ± SD:** NR **Sex:** Girls only **Sample size:** Baseline: n = 160 [participating: n = 138, IG: n = 68 (89.4%) & CG: n = 70 (89.7%)] **School/s:** 2 public intermediate schools
Choudhury et al. (2018) [38] Qatar	**Design:** Pre-post study **Duration:** 5 months	**Age:** 7–12 years **Mean age ± SD:** 9.1 ± 1.2 years **Sex:** Boys and girls **Sample size:** Baseline: n = 335 (boys: n = 186 & girls: n = 149) and follow-up: n = 278, (83.3%) (boys: n = 148 & girls: n = 130) **School/s:** 1 public primary school
Elfaki et al. (2020) [39] Saudi Arabia	**Design:** Cluster RCT **Duration:** 6 months	**Age:** Students aged 12–15 years **Mean age ± SD:** NR **Sex:** Girls only **Sample size:** n = 565 **School/s:** 4 public schools (grades 2 & 5)
Hefni [45] (2017) Saudi Arabia	**Design:** Pre-post study **Duration:** 3 months	**Age:** 9–16 years **Mean age ± SD:** 11 ± 1.86 years **Sex:** Girls only **Sample size:** n = 90 (9–10 years, n = 31; 11–12 years, n = 24; 13–16 years, n = 35) **School/s:** 1 public school
Hussein (2017) [40] UAE	**Design:** Pre-post study **Duration:** 6 months	**Age:** 5–18 years **Mean age ± SD:** NR **Sex:** Boys and girls **Sample size:** n = 2890 (school 1) & n = 1077 (school 2) **School/s:** 2 private schools
Kutbi (2019) [41] Saudi Arabia	**Design:** Cluster RCT **Duration:** 6 months	**Age:** 11–19 years **Mean age ± SD:** 14.45 ± 2.32 years **Sex:** Boys only **Sample size:** 5 (2 primary, 2 intermediate & 1 high) schools **School/s:** n = 148 [primary schools n = 38 (25%); intermediate schools n = 62 (41.9%); & High schools n = 49 (33.1%)]
Shama and Abdou (2009) [42] Oman	**Design:** Post-test study **Duration:** 12 months	**Age:** 13–16 years **Mean age ± SD:** NR **Sex:** Boys and girls **Sample size:** n = 1535: [HPS n = 752 (boys: n = 312 &, girls: n = 440) & CS n = 783 (boys: n = 299, girls: n = 484)] **School/s:** 30 schools [(HPS: n = 15 & CS: n = 15): grades 8 & 9]

*Abbreviations*: *CG* Control group, *CS* Conventional school, *IG* Intervention group, *HPS* Health-promoting school, *NR* Not reported, *RCT* Randomised control trial, *SD* Standard deviation, *UAE* United Arab Emirates

### Study characteristics

As illustrated in Table 1, six studies were conducted in Saudi Arabia [35, 37, 39, 41, 44, 45], two in Kuwait [36, 43], and one each in Qatar [38], the UAE [40] and Oman [42]. All included studies were published between 2017 and 2022 except the one by Shama and Abdou [42] in Oman, which was published in 2009. Out of the eleven studies, six were randomised controlled trials [35–37, 39, 41, 43], four were pre-post studies [38, 40, 44, 45] and one used a static group comparison design [42]. Six studies were purely quantitative [35, 39–42, 45], and two adopted a mixed methods design [43, 44].

### Participant characteristics and settings

The sample sizes ranged from 28 in an RCT [35] to 3,967 participants across two schools in a pre-post study [40]. The age of the participants ranged from five to nineteen years. In six studies, the study participants were girls only [35, 37, 39, 43–45], while four studies had male and female students [36, 38, 40, 42] and one study had only male participants [41]. In six studies, the participants were students from intermediate and/or secondary schools [35, 37, 39, 42–44], two studies had primary school students [36, 38], one had students from primary, intermediate, and secondary schools [41], while the school education stage was not clearly reported in two studies [40, 45]. The number of schools participating in the studies ranged from one [38, 43–45] to thirty [42]. The interventions were either only conducted at public schools [36–39, 43–45], only at private schools [40], at public and private schools [36], while two studies did not report the school type [41, 42]. The study in Oman by Shama and Abdou [42] compared conventional schools with health-promoting schools, which have three main components: health education, health services, and a healthy environment. Intervention components and outcomes of the included eleven studies are detailed in Table 2.

**Table 2:** Intervention components and outcomes of included studies (n = 11)

Reference	Intervention components	Main findings	Theory-guided
Al-Failakawi (2017) [43] Kuwait	***Education:*** The researcher delivered six educational sessions (45 min. each) once/month about various topics related to health behaviours. The study employed a semi-structured health-related behaviours and attitudes questionnaire (HRBQ) to gather data on multiple behaviours, including physical activity, eating habits, use of medications and other drugs, tobacco smoking, and UVR exposure/sun protection. Additionally, a dietary questionnaire was developed to assess eating habits and the frequency of food intake	***Anthropometrics:*** NS decrease in body weight measures: BMI (*p* = 0.6), Body fat (*p* = 0.603), and WC (*p* = 0.8) ***Diet:*** IG had improved a range of dietary practices: breakfast/week (*p* = 0.04), dairy intake/day (*p* = 0.02), and water intake/day (*p* = 0.003) ***Physical activity:*** IG had a significant increase in the total walking time during breaks (*p* < 0.0005), in the total time of moderate exercise/sports (*p* = 0.04) & in transport walking (*p* = 0.02). However, there was no significant interaction between groups and in time spent in moderate intensity housework (*p* = 0.3) or in vigorous physical activity (*p* = 0.2) ***Sedentary behaviour:*** IG had less sedentary behaviour (NS) during breaks (*p* = 0.2) and had a significant decrease in the frequency of elevator use (*p* = 0.02) ***Other outcomes:*** The IG had a significant increase in health knowledge of each topic compared to the CG (*p* < 0.0005)	SCT
Al-Jaaly (2017) [35] Saudi Arabia	***Education:*** The participants were instructed on changing their dietary and physical activity behaviours, increasing the duration and types of different physical activities, increasing consumption of fruit and vegetables, and reducing the intake of sugar-sweetened beverages ***Persuasion:*** Changes in the perceptions of the intervention group were observed, such as the perceptions of being healthy and the importance of performing physical activity	***Anthropometrics:*** Using the Saudi growth chart, the prevalence of overweight was (15.4% & 16.2%) & obesity (12% & 4.4%) in the public and private school, respectively. WC for 69% of girls in the private schools scored ≥ 90th percentile compared to 45% in public schools. There was a significant decrease in BMI in the intervention group (*p* = 0.009) ***Diet:*** No significant differences among the IG (pre & post-intervention) in all dietary behaviours in terms of the effect of peers & families on meal size (*p* = 0.02), meal skip (*p* = 1.00), purchasing from the school canteen (*p* = 0.3) or following a specific diet (*p* = 0.2). No significant differences between the IG and the CG in all previously mentioned behaviours ***Physical activity:*** Significant increase in self-reported physical activity outside school (*p* = 0.005) ***Other outcomes:*** Not relevant to our research question	NM
Allafi (2020) [36] Kuwait	***Education:*** The feedback group (FB) received information about the function of the pedometer. In contrast, the feedback with rewards group (FB + R) received information about the pedometer function and was asked to achieve a milestone of 3000 step counts to earn rewards ***Incentivisation:*** The FB + R group was rewarded with stickers for achieving the step count milestone, serving as an incentive to encourage physical activity ***Coercion:*** Participants were randomly assigned to one of the three groups (FB, FB + R, C) to minimise bias and ensure equal distribution of participants. The study’s control group (C) received no information about the pedometer function or rewards ***Training:*** Participants were given pedometers and were instructed to wear the pedometers throughout the physical activity sessions and to achieve a certain number of step counts	***Anthropometrics:*** NS difference between boys and girls in the average BMI (*p* = 0.15) ***Physical activity:*** The average step counts were (2091 ± 483) for CG, (2655 ± 577) for FB, and (3429 ± 458) for FB + R. Significant increase in the average step counts among FB + R compared with CG as well as among FB group compared with CG (*p* < 0.001)	NM
Al-Mughamisi (2021) [44] Saudi Arabia	***Training:*** Students conducted canteen food-scaping to identify the current provisions and assess healthfulness. Intervention-modelling workshops, using semi-structured questionnaires, were executed for stakeholders (10 students, 11 MoE and school staff) to identify the content and mode of delivery of the canteen intervention ***Enablement:*** The study highlights the importance of developing partnerships with relevant stakeholders to assess the acceptability of the intervention and foster communication ***Modelling:*** Students who engaged with the intervention may influence other peers to do so ***Environmental restructuring:*** The school food environment was modified, enabling more healthful food choices ***Restrictions:*** Restricting access to unhealthy foods was applied as part of the environmental changes	***Diet:*** Significant increase in students who consumed healthy food options from 6 to 34% (*p* = 0.001), healthy drink options from 11 to 32% (*p* = 0.01)	SEM
Bahathig (2022) [37] Saudi Arabia	***Education:*** Several information sessions included discussions, educating the participants about nutrition, physical activity, and body image perception. The participants were also asked to provide self-reported data at three-time points: pre-intervention, post-intervention, and follow-up ***Training:*** Participants were engaged in practical activities related to nutrition, physical activity, and body image perception, including matching food groups with the “Healthy Food Palm”, comparing food labels, warm-up exercises, skipping, walking, dancing using the hoop and jogging ***Enablement:*** Various teaching aids such as PowerPoint presentations, booklets, games, papers and cards, school boards, group discussions, and stickers were used to enhance the learning experience and engage the participants in the intervention	***Anthropometrics:*** NS difference in BAZ (*p* = 0.51) or WC (*p* > 0.7) between all pre-, post-intervention and follow-up values ***Physical activity:*** Significant difference in physical activity between pre-intervention and post-intervention (*p* < 0.001), between pre-intervention and follow-up (*p* < 0.001), and between post-intervention and follow-up (*p* = 0.05) ***Sedentary behaviour:*** Significant reduction in both screen time and the total SB on both weekdays and weekends between pre- and post-intervention & between post-intervention and follow-up (*p* < 0.001)	
Choudhary (2018) [38] Qatar	***Education:*** Students were provided with regular face-to-face consultation and feedback by the school nurse and catering staff concerning food selections in the cafeteria, aimed at encouraging students to choose healthier options. Students were also educated about healthy eating habits and portion sizes through various means, including an informational DVD watched during class. Factual nutritional information and advice were provided in the campaign to increase students’ knowledge about healthy food selection. Information and recipe cards were also prepared to educate students about healthy food choices ***Incentivisation:*** Students received stamps in a book when they chose a healthy option in the cafeteria. After obtaining a pre-specified number of stamps, students were awarded a badge to motivate and reinforce students’ healthy eating behaviours ***Environmental restructuring:*** The school cafeteria was redesigned with colourful posters providing information about the benefits of macronutrients and the importance of consuming fruits and vegetables	***Anthropometrics:*** NS increase in obesity (*p* = 0.51) and NS decrease in overweight (*p* = 0.15). NS change in BAZ (*p* = 0.22) and no change in WC (*p* = 0.11) or waist-to-hip ratio. As measured by bioelectrical impedance analysis, significant increase in fat mass (*p* = 0.003), muscle mass (*p* < 0.001) and fat free mass (*p* < 0.001). Significant increases in hip and neck circumferences (*p* < 0.001) ***Diet:*** Significant decrease in intake of energy drinks (*p* = 0.05) and rice (*p* < 0.001). NS increase in fruits and vegetables’ consumption and NS decrease in eating unhealthy foods ***Physical activity:*** Significant increase in time spent in light activity (*p* < 0.001) and in moderate (*p* = 0.031) ***Sedentary behaviour:*** NS change in SB (*p* = 0.98)	NM
Elfaki (2020) [39] Saudi Arabia	***Education:*** Health education classes about healthy eating and physical activity were provided to the students. Messages and materials were delivered to students’ parents. The one-day counselling session, including lectures and open discussions, role-playing, games, and questions, was given to convey knowledge to all students and teachers, influence students’ attitudes and behaviours, and assess student’s knowledge and practice toward their healthy lifestyle ***Training:*** A morning session in the form of physical exercise training was conducted before class. An individual intervention plan was prepared for overweight and obese students	***Anthropometrics:*** NS decrease in the prevalence of obesity (*p* = 0.06) and no change in the prevalence of overweight among students ***Diet:*** Significant decrease in weekly fast food intake (*p* < 0.001), bread intake (*p* = 0.01) and in the number of snacks between meals (*p* < 0.001). No significant difference in eating fruits and vegetables. Significant increase in consumption of water (*p* < 0.05) and soft drinks ***Physical activity:*** Significant increase in walking at least 10 min/day (*p* < 0.001) and in moderate physical activity (*p* = 0.02). However, NS increase in vigorous-intensity physical activity	NM
Hefni (2017) [45] Saudi Arabia	***Education:*** Education and information about nutrition and obesity-related knowledge were provided to students and their parents. Monitoring and tracking behaviour changes through self-reported questionnaires were completed by students and their parents. Booklets about obesity risk and the diet plan table were prepared, educating students and their parents ***Persuasion:*** Participants were encouraged to choose healthy foods and the importance of a balanced diet. They were promoted to regular physical activity and reduced sedentary behaviour. Participants were encouraged to consume the recommended amounts of fruits and vegetables. They were fostered for positive attitudes towards behaviour change through cultural sensitivity and tailored interventions. Relevant pictures to visualise excellent and lousy eating habits of overweight or obese, including sedentary behaviour and a sugar-dense diet ***Training:*** Interactive session (20 min): three activities or workshops, namely healthy eating and tasting, healthy eating colouring, and healthy quizzes and games ***Enablement:*** Parents were involved in the intervention by asking them to respond to questionnaires that validated their children’s responses	***Anthropometrics:*** The prevalence of girls with normal weight increased and with overweight and obesity decreased. NS significant differences in the average BMI ***Diet:*** Self-reported and parental-reported intake of unhealthy food products (fast food, soft drinks, energy drinks and snacks) decreased. Consumption of healthy food products (meat, beans or nuts, bread or cereals, vegetables, fruits and dairy products) increased ***Physical activity:*** Significant increase in vigorous physical activity (*p* < 0.001) ***Sedentary behaviour:*** The number of participants with more than 1 h per day watching TV, on the computer and playing video games decreased	SCT
Hussein (2017) [40] United Arab of Emirates	***Education:*** Health education sessions, awareness sessions, and competitions aiming to educate and raise students’ awareness about the importance of healthy behaviours and reinforcement of behaviour change. Families were given parents’ guidelines to handle obesity within the family environment ***Training:*** Competitions for healthy food selection. Physicians, nurses, and nutritionists were trained on childhood obesity management guidelines. Healthcare professionals were equipped with the knowledge and skills necessary to address childhood obesity ***Enablement:*** Different strategies were implemented based on the family’s preference. Some families chose to work with a family physician in the private sector, while others preferred to work with a school nurse and school clinic ***Environmental restructuring:*** In the intervention, foods in the schools’ canteens were classified according to their nutrient value and labelled as healthy and unhealthy foods using different colours (green, yellow, and red)	***Anthropometrics:*** Decrease in the prevalence of overweight in both schools (15.9% and 15.4% pre-intervention – 15.6% & 14.7% post-intervention). Decrease in the prevalence of obesity in both schools (14.4% & 14.8% pre-intervention – 13.9% & 14.2% post-intervention)	NM
Kutbi (2019) [41] Saudi Arabia	***Education:*** An hour of health education session was provided in the first and fifth weeks. 5–10 min presentations about a task related to a healthy diet were conducted in the third and seventh weeks. Group counselling was delivered in the second and sixth weeks. The presentations prepared by the students were discussed in the fourth and eighth weeks	***Anthropometrics:*** NS difference in BMI (*p* = 0.15), in body fat percentage (*p* = 0.16) and in fat mass (*p* = 0.19), as measured by Tanita BC 418 segmental body composition analyser ***Diet:*** NS increase in daily consumption of vegetables (from 22.9% to 24.3%) and fruits (from 8.7% to 15.7%) and decrease in the intake of milk (from 37.7% to 27.1%) ***Physical activity:*** NS increase among the IG in the total METs between pre (2098.41 ± 1922.67) and post-intervention (2497.95 ± 2291.13). NS difference in total METs between the IG and the CG (2556.27 ± 2048.71). Among the IG, NS increase in the percentage of students who met the recommended > 1680 METs- min/week (from 47.1% up to 54.3%) ***Sedentary behaviour:*** NS difference between the IG and the CG in the time for watching TV (*p* = 0.58), computer use (*p* = 0.17), total screen time (*p* = 0.44) and sleeping time (*p* = 0.69)	SCT
Shama & Abdou (2009) [42] Oman	The specific behavioural change techniques or procedures used in the study are not mentioned explicitly in the provided context. However, multiple interventions assumed to be conducted in the health-promoting school (HPS) initiative, such as: ***Education:*** Health education on various topics related to physical activity, sedentary behaviours, and dietary nutrition was provided to the study’s participants ***Enablement:*** Community partnership with the HPS. Psychological support ***Environmental restructuring:*** School nutrition service and school physical environment	***Anthropometrics:*** Significant decrease in the prevalence of underweight and obesity while increase in the prevalence of overweight male students in the HPS, and NS difference in weight measurements among female students in the HPS and the CS ***Diet:*** NS increase in male and female students having breakfast 6–7 times/week. Significant increase in vegetable intake among girls at HPS (*p* < 0.05). NS decrease in consumption of fast food and soft drinks among male students in HPS, no difference between female students in HPS and CS in consumption of fast food and soft drinks	NM

*Acronyms*: *BAZ* BMI-for-age z-score, *BW* Body weight, *CG* Control group, *FB* Feedback group, *FB* + *R* Feedback plus rewards group, *HDL* High density lipoprotein, *HPS* Health promoting schools, *IG* Intervention group, *METs* Metabolic equivalents, *MoE* Ministry of Education, *NS* Non-significant, *NM* Not mentioned, *PA* Physical activity, *SB* Sedentary behaviour, *SCT* Social cognitive theory, *SEM* Social ecological model, *TG* Triglycerides, *WC* Waist circumference

### Intervention components

The intervention duration ranged between one month [35] and one year [42]. However, it was not clearly reported in one study [44] and not reported at all in another study [36]. To change participants’ behaviour as per the BCW, intervention components in our systematic review were reported under nine intervention functions, including education; persuasion; incentivisation; coercion; training; enablement; modelling; environmental restructuring; and restrictions.

Participants in all included studies [35–41, 43–45] were educated on various topics related to nutrition, physical activity, or health, including the study by Shama and Abdou [42], where health education was a component of the health-promoting schools initiative. Different methods of educational interventions were implemented by providing factual nutritional information and advice (e.g. nutritional posters and cards, recipe cards) to the students and their families [38], instructions for changing nutritional and physical activity behaviours (e.g. increasing fruit and vegetable intake and reducing the intake of sugar sweetened beverages, receiving information about the function of the pedometer) [35, 36], health education and awareness workshops and sessions on diet, physical activity, and obesity risk factors [37, 40, 41, 43, 44], and counselling sessions [38, 39].

Out of the eleven studies, only two [35, 45] used the persuasion function to change participants’ behaviours. There were changes in the perceptions of the intervention group in one study [35], and positive attitudes were reported in the other study [45]. Similarly, incentivisations were reported in two studies [36, 38]. In Allafi’s study [36], the FB + R group was provided with rewards in the form of stickers for achieving the step count milestone. However, in Choudhury’s study [38], participants received stamps in a book when they chose a healthy option in the cafeteria and were awarded a badge at the end, to motivate and reinforce healthy eating behaviours.

Interventions via training were reported in six studies [36, 37, 39, 42, 44, 45], where the participants were engaged in various practical activities related to nutrition, physical activity, and body image perception (see Table 2). Environmental restructuring was reported in three studies in the forms of food labelling and promotion of healthy diets at the school cafeteria [40] and redesigning the school canteen, which included posters and leaflets about healthy diets [38] and was a component of the health-promoting school initiative in the study conducted in Oman by Shama and Abdou [42].

Five studies [37, 40, 42, 44, 45] reported changes in participants’ behaviours through partnerships with stakeholders, including students’ parents [40, 42, 44, 45] or by enabling various teaching aids such as PowerPoint presentations, booklets, games, papers and cards, school boards, group discussions, and stickers [37]. The restriction function of the BCW to change students’ behaviours was reported only in the study conducted by Al-Mughamisi [44], where restricting access to unhealthy food was applied as a part of the environmental changes.

In addition to targeting the school students, three interventions [38, 40, 44] also targeted the teachers and other school staff, while another five studies involved the students’ parents [35–37, 39, 44].

### Intervention outcomes

The outcomes of interest were weight-related measures, dietary behaviour, physical activity, and sedentary behaviour. All studies reported weight-related outcomes, such as a change in BMI, BMI-for-age z-score (BAZ), the prevalence of overweight or obesity, body weight perception, body fat percentage, body fat mass, waist circumference, or waist-to-hip ratio, except one study [44]. Most of the included studies [35, 38, 39, 41–45] reported changes related to dietary intake and/or behaviour. Eight studies reported outcomes related to physical activity [35–39, 41–43, 45], and five reported sedentary behaviour outcomes [35, 37, 41, 43, 45].

#### Changes in weight-related measures

One study found a significant decrease in BMI, based on the Saudi growth chart [35], while four studies reported either a non-significant difference or no chage in BMI [36, 41, 43, 45]. Two studies reported a non-significant reduction in BAZ [37, 38]. Three studies [38–40] reported the prevalence of overweight and obesity as a weight-related measure, with mixed results. Elfaki et al. [39] reported a borderline significant decrease in the prevalence of obesity (*p* = 0.064), and Choudhury et al. [39] found a non-significant decrease in overweight (*p* = 0.15). However, Hussein [40] reported a reduction in the prevalence of obesity and overweight across the two schools under study (*p* values not provided). A range of statistics of participants’ anthropometrics and weight change was reported in the included studies, such as numbers, percentages, means, standard deviations, and odds ratios. Participants’ weight change was presented in terms of the number and percentage in four studies [35, 37, 42, 43], while the prevalence of overweight and obesity was presented using the percentages in one study [40]. In comparison, five other studies reported weight change using means and standard deviations [36, 38, 39, 41, 45]. Means and standard deviations were reported for diverse anthropometrics, such as participants’ weight, height, BMI, BMI z-scores, fat mass, waist circumference, and waist-to-hip ratio in six studies [36, 38, 39, 41, 43, 45]. However, none of the included studies reported odds ratio for the association between body weight and other variables of interest, except in one study [44].

#### Changes in dietary behaviour

All studies reported different outcomes related to dietary behaviours, except three studies [36, 37, 40]. Al-Failakawi [43] reported a significant increase in dietary knowledge (*p* < 0.0005) with a significant increase in the percentage of students who had breakfast (*p* < 0.004), dairy intake per week (*p* < 0.02), and water intake per week (*p* = 0.003). There was a significant increase in students considering themselves to have a healthy diet (*p* = 0.03) [35] or those who had access to healthy food (*p* < 0.001) and drinks (*p* < 0.01) [44]. Hefni [45] also found a reduction in the consumption of unhealthy food and an increase in healthy food intake (*p*-values not provided). Elfaki et al. [39] reported a significant reduction in the intake of fast food (*p* < 0.001) and Shama and Abdou [42] reported a significant reduction in fast food and soft drink intake (*p* < 0.05). Choudhury et al. [38] reported a borderline significant reduction in energy drink intake (*p* = 0.05) and a significant decrease in rice intake (*p* = 0.01). Shama and Abdou [42] reported a significant increase in the proportion of participants that had breakfast (*p* < 0.05) and in vegetable intake (*p* < 0.05) among girls in health-promoting schools. Kutbi and colleagues [41] also reported similar findings with increased vegetable and fruit consumption.

#### Changes in physical activity

Eight interventions targeted physical activity [35–39, 41, 43, 45] with various indicators to measure the outcomes while the other three studies [40, 42, 44] did not report any physical activity outcomes. Seven studies [35–39, 43, 45] reported a significant change in at least one physical activity outcome except one study [41], where Kutbi et al. found a non-significant increase in the total metabolic equivalents (METs) among the intervention group between pre- and post-intervention, (2098.41 ± 1922.67 and 2497.95 ± 2291.13, respectively). Elfaki and colleagues [39] reported increases in the number of days with walking for more than 10 min (*p* < 0.001) and time engaged in moderate physical activity during the intervention (*p* < 0.001). Two studies reported significant increases in daily light-intensity activity [38, 43], energy expenditure measured by accelerometer (*p* < 0.02) [43], or performing any kind of physical activity outside school (*p* = 0.003) [35]. A study in Qatar [38] found no significant changes in moderate-to-vigorous activity, while a study from Saudi Arabia [39] reported a significant increase in moderate physical activity.

#### Changes in sedentary behaviour

Five studies evaluated intervention effects on sedentary behaviour [35, 37, 41, 43, 45]. Al-Failakawi [43] reported a significant decrease in time spent in sedentary behaviour (*p* = 0.03) and elevator use (*p* < 0.023). Bahathig and Abu Saad [37] reported significant improvements in sedentary behaviours (*p* < 0.001) among the intervention group compared with the control group. Hefni [45] reported reduced time spent watching television, computer use and using smartphones (no *p*-values provided) and Kutbi et al. [41] reported non-significant differences between intervention and control groups for TV watching (*p* < 0.58), computer use (*p* < 0.17) and sleep time (*p* < 0.69). However, Al-Jaaly [35] found a non-significant influence of watching TV on students’ eating behaviours (*p* = 0.119).

### Quality of included studies

Based on the Effective Public Health Practice Project (EPHPP) quality assessment tool, the quality of two studies was rated as ‘moderate’ [41, 43], and ‘weak’ for the other nine [35–40, 42, 44, 45] (Table 3). The study by Hussein [40] was rated ‘weak’ in all six components of the assessment tool. All included studies were rated ‘weak’ in blinding, except for the study by Hefni [45]. Other ‘weak’ ratings were mainly due to selection bias [36, 40, 42], study design [35, 38, 40, 42, 44, 45], confounders [35–40, 42, 44, 45], data collection methods [35, 36, 40, 44], withdrawal and dropouts [35–37, 40, 42, 44].

**Table 3:** Methodological quality appraisal and effectiveness of interventions of included studies

Study author/s and date	Quality assessment using the EPHPP							Effectiveness of interventions			
	**Selection bias**	**Study design**	**Confounders**	**Blinding**	**Data collection methods**	**Withdrawal and dropouts**	**Overall quality score**	**Body weight**	**Dietary behaviour**	**Physical activity**	**Sedentary behaviour**
Al-Failakawi (2017) [43]	Strong	Strong	Moderate	Weak	Strong	Strong	**Moderate**	N	NR	E	E
Al-Jaaly (2017) [35]	Moderate	Weak	Weak	Weak	Weak	Weak	**Weak**	E	N	M	N
Allafi (2020) [36]	Weak	Moderate	Weak	Weak	Weak	Weak	**Weak**	N	NR	E	NR
Al-Mughamisi (2021) [44]	Moderate	Weak	Weak	Weak	Weak	Weak	**Weak**	NR	N	NR	NR
Bahathig & Abu Saad (2022) [37]	Moderate	Strong	Weak	Weak	Strong	Weak	**Weak**	N	NR	E	E
Choudhury et al. (2018) [38]	Strong	Weak	Weak	Weak	Strong	Strong	**Weak**	N	M	M	N
Elfaki et al. (2020) [39]	Moderate	Strong	Weak	Weak	Strong	Strong	**Weak**	N	E	E	NR
Hefni (2017) [45]	Strong	Weak	Weak	Moderate	Strong	Strong	**Weak**	N	E	E	E
Hussein (2017) [40]	Weak	Weak	Weak	Weak	Weak	Weak	**Weak**	E	NR	NR	NR
Kutbi et al. (2019) [41]	Moderate	Strong	Strong	Weak	Moderate	Strong	**Moderate**	N	N	N	N
Shama & Abdou (2009) [42]	Weak	Weak	Weak	Weak	Strong	Weak	**Weak**	E	E	NR	NR

*Acronyms*: *E* Effective, *M* Mixed effect, *N* Not effective, *NR* Not reported, *EPHPP* Effective Public Health Practice Projects Quality Assessment Tool for Quantitative Studies

## Discussion

This is the first systematic review that explores school-based weight-related interventions among children and adolescents in the GCC countries. Despite the high prevalence of childhood obesity in the six GCC countries [13, 14], we only found eleven intervention studies aimed at reducing obesity among school students. Similarly, a systematic review on promoting physical activity across all Arab-speaking countries reported that only 13 of the included 39 studies focused on participants between 5 and 19 years of age [33].

### Quality of included studies

The included studies were limited in their study designs in terms of sampling errors and participant allocation. Six of the included studies were randomized controlled trials (RCTs) [35–37, 39, 41, 43], which are generally considered robust for intervention evaluations [49], including school-based weight-related interventions [20]. However, several methodological weaknesses were noted among these studies. For instance, in one RCT [35], the sample size was notably small (n = 28), leading to concerns about statistical power. Additionally, this study and another RCT [39] had unequal numbers of participants in the intervention and control groups, potentially affecting the balance and comparability of these groups. These issues, beside others, such as unreported confounders and variations in study designs, collectively suggest that the overall strength of the study designs was weak or moderate at best. Such limitations should be carefully considered when interpreting the results of these interventions. The RCT by Al-Failakawi [43] had a large sample size (n = 128) with participants assigned equally among the control and intervention groups, however, the study is limited in terms of blinding. Four interventions were pre-post studies [38, 40, 44, 45], and one used a post-test design [42], which threatens internal validity in terms of selection bias. Furthermore, only two studies [41, 43] included in this review reported confounders. In addition to these methodological concerns, the lack of detailed reporting on confounders and other potential statistical errors (such as improper use of *p*-values or effect sizes) further limits our ability to accurately gauge the interventions’ true effects, in line with the concerns raised by Brown et al. [50]. These methodological limitations warrant more rigorous study designs for future school-based interventions. Habib-Mourad et al. [51] pointed out that weight-related interventions involving children would require large sample sizes and sufficient follow-up periods to observe significant changes in the outcomes. The studies included in this review had sample sizes between 28 and 2890 and follow-up times between 1 and 12 months. The finding regarding the methodological limitations of the included studies is in line with another systematic review on obesity interventions for adults in the GCC states, which found that most evaluations of interventions did not have control groups [32].

### Theoretical concepts and frameworks

Various intervention’s components were used in the studies included in this review, typically in multi-component weight-related interventions in school settings [52]. However, the development of the intervention components was not explicitly discussed in some of the included journal articles. Previous studies that reported successful outcomes, such as weight-related measures, physical activity, and nutrition behaviours, adopted one or more theoretical frameworks [52, 53]. None of the journal articles included in our systematic review reported any theoretical underpinning except the study conducted by Kutbi and colleagues [41], which was based on the social cognitive theory. However, one of the doctoral theses was based on the social-ecological model [44], and the other two were based on social cognitive theory [43, 45]. Particularly in school settings, where multiple stakeholders can work together to achieve a common goal in health promotion interventions, it would be crucial to consider theoretical concepts in the design of the studies [24]. Theory-driven interventions are also important for translating evidence into practice and in making relevant decisions for applying the intervention components in practice [54]. Our review found that a variety of theoretical frameworks underpinned the interventions, emphasising the importance of a multifaceted approach to obesity intervention. These theories highlight the significance of multiple and combined factors in shaping health outcomes. However, the inconsistent application of these theories across studies suggests a need for more robust theoretical grounding in future research.

### Interventional aspects

Despite the limitations of the included interventions, this review suggests that though there is some potential, the overall evidence supporting the effectiveness of school-based interventions in addressing obesity is mixed and warrants cautious interpretation. This is consistent with other reviews of similar interventions in high-income countries [55, 56] and low to middle-income countries [57, 58].

Studies have revealed that the success of interventions could potentially be attributable to the school’s environmental support in terms of finance, incentives, and applying weight management-related policies [56–59], highlighting the importance of multiple concomitant approaches to counteract obesity or its linked factors among students at schools. Environmental components were only included in three of the school-based interventions [38, 40, 41], indicating more research is required in this area. Considering the social context of the school environment, facilitators and barriers are crucial to determine the success or failure of weight-related interventions [33, 60].

There was considerable variation in the outcomes of the included studies, with some showing improvements in students’ anthropometrics in terms of reducing the prevalence of overweight or obesity, and decreases in BMI or waist circumference, while others did not, highlighting the need for a cautious interpretation of these results. Students’ weight outcomes were improved in some of the included studies [35, 37, 39, 40, 42]. Some systematic reviews in other parts of the world have suggested a range of outcomes, from mild to significant effectiveness of school-based interventions for addressing childhood obesity, particularly if they were long enough, used multiple components, and had parental involvement in delivering the intervention [58, 59]. However, it is important to note that results vary widely, and some studies, including a notable Cochrane Review, report limited effectiveness [61], two meta-analyses conducted by Kanekar and Sharma [62], covering studies from the USA and UK and the other by Harris et al. [63], including twelve studies from the USA, three from Canada and one each from Australia, Chile, and Sweden. Accordingly, there is various evidence that school-based interventions can improve students’ weight status.

The studies in this review demonstrated significant improvements in dietary habits, such as decreased energy and soft drink consumption and increased intake of fruits and vegetables, water, fish, and dairy. These findings align with other similar systematic reviews about school-based weight-related interventions from the USA and Europe [64, 65]. Overall, this suggests that school-based interventions in the GCC countries may improve students’ dietary habits and eating behaviours.

The intervention outcomes of the included studies suggest that physical activity and sedentary behaviour can potentially be improved with school-based interventions in the Gulf countries, which is consistent with studies on school students from other parts of the world [66, 67]. The included studies used various measures for physical activity and sedentary behaviour, which did not allow for direct comparisons across the studies. This calls for standardised, valid, and reliable measurements to improve evidence-based health promotion [33].

### Strengths and limitations

A key strength of this study is that to the best of our knowledge, it is the first comprehensive systematic review of school-based weight-related interventions in the GCC countries, a region with one of the highest obesity rates in the world. This review fills a significant gap by providing information on the participants, characteristics, components, outcomes and strengths and limitations of the interventions. A further strength of this systematic review is that we utilised a rigorous and comprehensive study design, including PROSPERO registration, following the PRISMA statement, and using the EPHPP tool for quality appraisal of the interventions. However, including only studies published in English is a limitation of our systematic review, potentially introducing language bias, although scientific studies and policy documents from the GCC countries are usually published in English rather than Arabic. An additional limitation was the preclusion of a meta-analysis due to the heterogeneity of the included studies in terms of study design and intervention components.

## Conclusion

Despite the methodological limitations of the included studies, this systematic review has identified important insights into school-based interventions for addressing childhood obesity in the GCC countries. A range of intervention strategies were reported, with a notable emphasis on multi-component approaches. The social-ecological model and social-cognitive theory were the conceptual frameworks commonly employed. Various study designs were reported, with several randomised controlled trials providing the highest level of evidence, albeit with limitations such as small sample sizes and unreported confounders. Significantly, these studies have provided tentative evidence as to the potential of school-based interventions to positively impact students’ weight status, dietary habits, physical activity levels, and sedentary behaviours, which will support evidence-based health promotion to address the obesity epidemic in the GCC countries. These findings emphasise the need for continued research with more rigorous, theory-based studies, particularly those that address the identified methodological gaps and contribute to developing effective, evidence-based strategies to combat childhood obesity in the GCC region. Policy initiatives that encourage and support the implementation of well-designed RCTs in schools are also highly recommended to evaluate the effectiveness of these interventions.

## Data Availability

Not applicable.

## Abbreviations

BAZ: BMI-for-age z-score
BCW: Behaviour Change Wheel
BMI: Body Mass Index
EPHPP: Effective Public Health Practice Project
GCC: Gulf Cooperation Council
PRISMA: Preferred Reporting Items for Systematic Reviews and Meta-Analysis
WHO: World Health Organization
